# Identification of Early Symptoms Associated with Subsequent Immune-related Adverse Events in the I-SPY clinical trial

**DOI:** 10.21203/rs.3.rs-8689063/v1

**Published:** 2026-02-11

**Authors:** Amrita Basu, Saumya Umashankar, Michelle Melisko, Ritu Roy, Christina Yau, Lajos Pusztai, A. Jo Chien, Minetta Liu, Hyo Han, Hatem Soliman, Claudine Isaacs, Rebecca Shatsky, Erica Stringer-Reasor, Patricia Robinson, Kay Yeung, Kevin Kalinsky, Alexandra Thomas, Errol Philip, Mina Musthafa, Gillian Hirst, Adam Asare, Angela DeMichele, Laura van’t Veer, Douglas Yee, Nola Hylton, Adam Olshen, Jane Perlmutter, Ronald N. Cohen, Zoe Quandt, Laura Esserman, Dawn Hershman, Hope Rugo, Rita Nanda

**Affiliations:** University of California, San Francisco; University of California, San Francisco; University of California, San Francisco; University of California, San Francisco; University of California, San Francisco; Yale University; University of California, San Francisco; Mayo Clinic; Moffitt Cancer Center; Moffitt Cancer Center; Georgetown University; University of California, San Diego; University of Alabama at Birmingham; Loyola University Chicago; University of California, San Diego; Emory University; Wake Forest University; University of California, San Francisco; University of California, San Francisco; University of California, San Francisco; University of California, San Francisco; University of Pennsylvania; University of California, San Francisco; University of Minnesota; University of California, San Francisco; University of California, San Francisco; Gemini Group; University of Chicago; University of California, San Francisco; University of California, San Francisco; Columbia University; University of California, San Francisco; University of Chicago

**Keywords:** breast cancer, adverse events, immunotherapy, hypothyroidism, adrenal insufficiency, hypophysitis

## Abstract

**Background:**

Immune checkpoint inhibitors can result in serious, long-lasting immune-related adverse events (irAEs). Early identification of symptoms predictive of irAEs could enhance monitoring and timely intervention. This study assessed whether symptoms within the first 8 weeks of treatment could predict subsequent development of immune-related adrenal insufficiency(AI) or hypothyroidism.

**Methods:**

This retrospective cohort study analyzed prospectively collected data from the I-SPY2 trial, a phase 2 platform trial for high-risk stage II/III breast cancer across 30 U.S. sites. The cohort included 482 women treated with experimental immunotherapy agents concurrent with weekly paclitaxel neoadjuvant chemotherapy. The primary outcomes were grade ≥ 1 hypothyroidism or AI, adjudicated by an independent safety group, up to 1-year post-treatment. Symptoms and irAEs were assessed using the Common Terminology Criteria for Adverse Events. Symptom burden was quantified as area under the curve (AUC) based on symptom grade and duration. Predictive modeling was performed using logistic regression and ROC analysis; symptom enrichment between cases and controls was evaluated using Fisher’s exact tests.

**Results:**

Among 482 participants, 107 (22.2%) developed irAEs, with hypothyroidism (n = 61, 12.7%) occurring more frequently than AI (n = 38, 7.9%) at medians of 99 and 105 days from treatment initiation, respectively. Symptom enrichment analysis identified early predictive symptoms. Fatigue (17.2% vs 6.8%, p = 0.011) and rash (20.7% vs 7.8%, p = 0.0037) were predictive of hypothyroidism, while diarrhea (45.9% vs 31%, p = 0.048), constipation (5.4% vs 0.2%, p = 0.018), and taste changes (5.4% vs 0.5%, p = 0.034) were associated with AI. A predictive model demonstrated moderate performance (AUC 0.65 for AI, p < 0.0001; AUC 0.61 for hypothyroidism, p = 0.012). Model accuracy in an external validation cohort was 72.8% for AI and 74.7% for hypothyroidism.

**Conclusions:**

This study presents a predictive framework to identify patients at risk for adrenal insufficiency and hypothyroidism as irAEs, enabling personalized care and proactive intervention to improve treatment outcomes and safety.

## Background

Recent advances in cancer immunotherapy, including the advent of immune checkpoint inhibitors (ICIs), have revolutionized cancer treatment, providing survival benefits across an array of cancer types, including breast cancer[1, 2]. ICIs harness patients’ adaptive immune system to treat cancer by antagonizing the receptors responsible for dampening the effective activity of immune cells and their ability to identify and remove tumor cells[1].

For breast cancer, pembrolizumab is approved in both early and metastatic triple-negative disease[3–5]. In addition, early trials have shown promising results of ICIs in early-stage hormone receptor-positive breast cancers[6–10].

ICIs are associated with immune related adverse events (irAE) that are often long-term[11–14]. These likely have multiple etiologies, leading to a broad range of autoimmune syndromes including potentially life-threatening events[15].

A systemic review by Balibegloo and colleagues[1] reported that approximately 12% of patients experienced any grade hypothyroidism, and about 1% of patients developed any grade adrenal insufficiency (AI), while being treated with PD-1 or PD-L1 inhibitors in the metastatic breast cancer setting. However, their incidence is higher in the adjuvant or neoadjuvant setting where these drugs are part of the standard of care for some tumor types[16]. Although the incidence of grade 3 or higher endocrine toxicity is low, even lower grade toxicity is nearly always permanent[15, 17, 18] and requires life-long medical management[19].

Identification of patients at higher risk of developing irAEs during treatment could offer an opportunity for early detection and intervention to prevent or minimize the impact of these conditions. For example, adjunctive immunomodulatory agents might help prevent or attenuate irAEs, and patients predicted to be at high risk for irAEs who experience rapid treatment response might consider discontinuing ICI therapy early.

We investigated the feasibility of early identification of patients at high risk of developing AI or hypothyroidism by analyzing the early symptom landscape. We used prospectively collected toxicity data from the ISPY-2 trial[16] and computed an overall symptom burden (OSB) score incorporating duration and severity of symptom to construct a predictive framework for clinicians to identify early symptoms that could forecast a later irAE, and conducted statistical enrichment analyses to uncover symptoms enriched in patients with and without the irAEs of interest.

## Methods

### Study Population

The I-SPY2 clinical trial is an ongoing, multicenter, open-label, response-adaptive phase 2 platform trial with multiple experimental arms evaluating new agents alone or in combination with weekly neoadjuvant paclitaxel followed by 4 additional cycles of doxorubicin/cyclophosphamide (AC) chemotherapy for women with high-risk stage II/III breast cancer (NCT01042379). Information regarding design, eligibility, and assessments has been previously reported[20].

This analysis used data from 2247 women enrolled between 2010–2022 at 30 clinical centers across the US. We identified 482 participants who received ICI therapy (± other experimental agents) concurrent with 12 cycles of weekly paclitaxel (Supplementary Figure S2) in 6 arms of I-SPY2. Information on treatment regimens is included in Supplementary Table 1.

#### Predictor Variables

32 symptoms that occurred on or before weeks 4, 6 and 8 were included as predictor variables. These were symptoms selected by a group of clinicians, researchers and patient advocates in the I-SPY patient outcomes working group as key symptoms to assess independently or for overall patient toxicity, quality of life or symptom burden. Adverse events in I-SPY2 are documented and graded using the Common Terminology Criteria for Adverse Events (CTCAEv4.0) weekly from treatment start till the study timepoint for symptom assessment (week 8). Start and end dates (if resolved) of the symptoms during this time frame were documented for each event. Fifty-five CTCAE categories were collapsed into 32 symptoms predictors (Supplementary Table 2). For symptoms graded on a 1–5 scale, grade 1 was excluded (deemed occasional/asymptomatic and did not interfere with activities of daily living). Grade 1 symptom events were included for dizziness, loss of fingernails or toenails, painful urination, and palpitations where events are not on a 5-point scale.

#### Outcomes

The primary outcome was the development of immune-related adverse AI (due to hypophysitis or primary adrenalitis) and hypothyroidism defined as grade 1 or higher at any time during the study, or through follow-up up until 1 year. These AEs were adjudicated by the I-SPY Safety Working Group, an external independent data safety and monitoring working group (CCSA), and endocrinologists (ZQ and RNC). Diagnoses for hypothyroidism were triggered through routine TSH and free T4 testing and symptom triggers. Diagnoses for AI were primarily symptom triggered, followed by cortisol and ACTH testing. These irAEs were selected because they are common and often become life-altering long-term irAEs. Groups considered for statistical comparison were patients that developed an irAE(s) of interest after treatment with experimental immunotherapy(case) vs those that did not(control).

### Statistical Analysis

A variable importance analysis was performed in two ways, first to characterize the association of OSB with the development of irAEs, and second, to understand sentinel symptoms associated with the development of irAEs of interest.

#### Overall Symptom Burden

For each patient, cumulative area under the curves (AUC) were calculated for each of the 32 symptoms based on their grade and duration at weeks 4, 6 and 8 as follows

$$\text{AUC}\left(\text{symptom}|\text{Week}\;\text{n}\right)=\sum_{\text{day}\;1}^{n\times 7}\text{AE}\;\text{grade}\times \text{days}\left(\text{in}\;\text{specified}\;\text{duration}\;\text{that}\;\text{AE}\;\text{persisted}\right)$$

Patients that developed an irAE of interest before the analysis timepoint (i.e. week 4, 6 or 8) were censored from the analysis of that timepoint for that outcome of interest.

Logistic regression was performed first between each different symptom AUC and the outcome of interest; those symptoms that had positive association and Wald test p-values < 0.2 were selected to calculate the OSB. The OSB is the sum of the selected symptoms. A logistic regression was then performed between the continuous OSB and outcome. For each time point, p-values were adjusted for multiple testing using the Bonferroni correction and an association was considered significant if the adjusted p-value was < 0.05. ROC curves were generated for analyses with significant association of the OSB with outcome.

The model developed above was validated in an external dataset comprising of 4 distinct arms in ISPY2 of 217 patients. Methods for additional statistical analyses and detailed validation methods are detailed in Supplementary Methods. All statistical analyses and visualizations were performed using R version 4.2.2.

## Results

### Patient Population

Among the I-SPY patients, 482 were randomized to one of the six arms including an immunotherapy component (median age = 47.3 years (range, 20–79 years)). 382 patients were white(79.3%) and 95 were non-white(19.7%). 78 patients reported Hispanic ethnicity (16.2%). Detailed demographics are summarized in Table 1.

**Figure 1:**
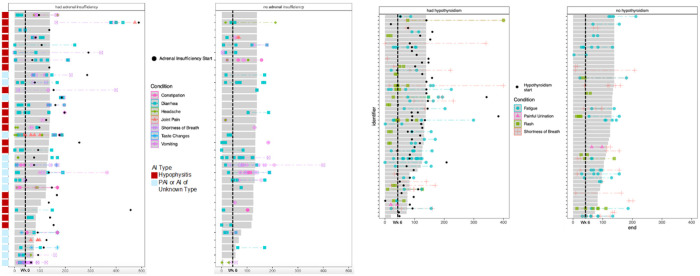
Swimmers plots showing the incidence, duration and co-occurrence of the symptom drivers symptoms in patients that had adrenal insufficiency vs. without (A), and in patients that had hypothyroidism vs. without (B). Each item on y axis represents a patient, with gray bars representing period of active treatment. Black points represent irAE start date

Among the cohort, hypothyroidism was more common(N = 61 or 12.7%) vs AI (38 or 7.9%). 21 of 61 hypothyroidism patients had hyperthyroidism an average of 4 weeks prior to hypothyroidism diagnosis. AI was most often attributed to confirmed or probable hypophysitis(65.8%), probable primary AI(2.6%), AI of unknown type(15.8%), and reported AI by site but unconfirmed at time of analysis by endocrinologist(15.8%)(Fig. 1A). No significant differences were observed in irAE incidence based on age, race or ethnicity.

#### Early Symptoms Predictive of Adrenal Insufficiency

AI was diagnosed at a median of 15 weeks(105 days) from treatment start(range = 6.6–41.6 weeks(46–291 days)), which was after completion of the immunotherapy phase and close to the end of all neoadjuvant chemotherapy(Supplementary Figure S2). One patient developed AI between weeks 4 and 6 and was censored from the weeks 6 and 8 analyses, while an additional patient developed hypothyroidism between weeks 6–8 and was censored from the week 8 analyses.

**Figure 2:**
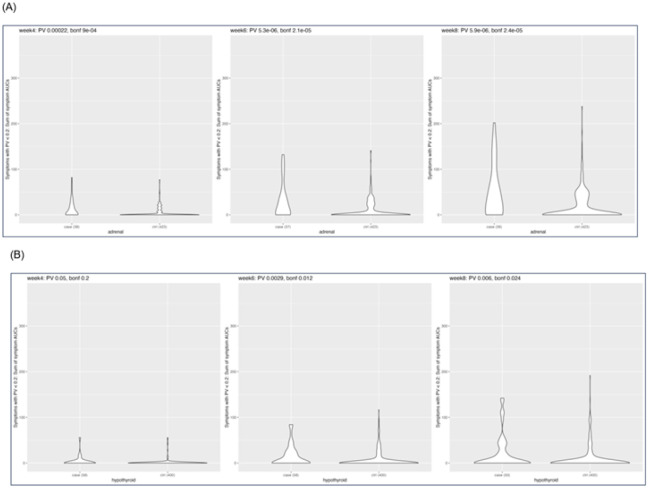
(A-B) Violin plots showing the difference in the overall symptom burden (OSB) between cases (irAE) and controls (no irAE) for adrenal insufficiency (A) and hypothyroidism (B). The p-value (PV) and Bonferroni adjusted p-value (bonf) for each model is provided above the plot.

OSB at week 6 was the timepoint most predictive of AI (adjusted p < 0.001) (Fig. 2A). Symptoms used to calculate OSB at week 6 were constipation, diarrhea, headache, joint pain, shortness of breath, taste changes, and vomiting (Supplementary Table 3). The trajectory of these symptoms as swimmers plots in patients with and without AI are shown on Fig. 1A. Results indicated that shortness of breath, for example, presented early and persisted through follow up, as did GI symptoms such as diarrhea. Patients who would later develop AI had multiple occurrences of bouts of diarrhea (instances > 2) compared to patients who did not. Patients who would later develop AI also had multiple co-occurring symptoms, the foremost of which was diarrhea and vomiting, while patients without AI rarely had these symptoms. These symptoms preceded the AI diagnoses by an average of 63 days (9 weeks).

#### Model Performance

A logistic regression was performed between the continuous OSB and outcome.

The Youden’s best OSB threshold was 14.5 with a sensitivity of 0.57, specificity of 0.74 and accuracy of 0.73 (Supplementary Table 6 and Supplementary Figure S1A). Mean OSB at week 4 of patients that developed AI was almost double that of patients that did not develop AI (mean(SD) cases = 13.66 (19.73) vs controls = 5.2 (10.92)), and this gap widened at weeks 6 (31.11(39.58) vs 10.6(10.67)) and 8 (49.81(57.79) vs 18.5(30.68)(Fig. 2A and Supplementary Table 3).

### Model Validation

In an external validation set of 217 patients receiving ICI with an oral paclitaxel backbone, 11 patients later developed AI. Patients were demographically comparable to the training set above (Supplementary Table 8). The OSB was computed as the sum AUCs of the symptoms mentioned above. Patients that later developed AI had a higher mean OSB at week 6 than patients that did not develop AI(44.6 vs 17.4, p = 0.02). Using the Youden’s best threshold above of 14.5, the model’s sensitivity was 54.6%, specificity was 73.8%, positive predictive value was 10%, negative predictive value was 96.8% and model accuracy was 72.8%.

### Longitudinal Symptom Enrichment

**Figure 3:**
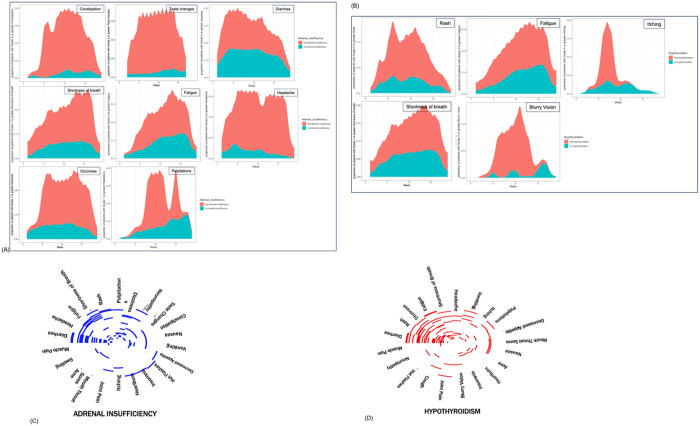
(A-B) Incidence of symptom presence over time. Symptoms with p value <0.2 are visualized in patients with adrenal insufficiency vs those without (A) and in patients with hypothyroidism vs those without (B). (C-D) Symptom co-occurrence heatmap for symptoms present prior to week 6 for patients with adrenal insufficiency (C) and hypothyroidism (D).

All symptoms were also analyzed as binary variables (present vs absent) at 4, 6, and 8 weeks (Supplementary Table 4). Unlike OSB, this analysis did not capture severity and duration of a given symptom and was performed so that we could identify additional symptoms that may be short lasting and more severe, or long lasting and less severe and may not be captured by the OSB analysis. Such an example was heart palpitations that was significant at the week 8 timepoint (adjusted p = 0.047) but receded p > 0.05 in the OSB analysis (Fig. 3A and Table 2). Interestingly, palpitations had similar rates of incidence in patients that developed AI compared to those that did not up until week 6, after which, patients that developed AI had a 4-fold higher rate of palpitations (8.3% in case vs. 1.9% in control).

In terms of symptoms that were overlapping between the OSB and binary analyses, prior to week 4, diarrhea was the most enriched symptom in patients that later developed AI, followed by constipation and shortness of breath (OR = 2.49, 23.03 and 3.25, adjusted p = 0.0069, 0.019 and 0.024 respectively). At week 4, diarrhea was present in 47.4% of the patients that later developed AI compared to 26.5% in patients who did not develop AI (Table 2). The difference in the proportions declined by week 6 (45.9% vs 31%) and was no longer significant at week 8 (47.2% vs 33.8%). Constipation was the only symptom that was significantly enriched at all three study timepoints. Constipation was present in twice as many patients who later developed AI than those without at weeks 4 and 6 (5.3–5.4% in cases vs < 1% in control) and in almost 8 times as many patients with AI by week 8 (8.3% vs. 0.7%). Patients that developed AI had almost twice the proportion of fatigue at weeks 4 (10.5% vs 4.3%) and 6 (16.2% vs 7.3%) compared to patients that did not develop AI, though this proportion nearly equalized by week 8 (13.9% vs 11.6%). Similarly, patients who later developed AI had almost three times the reported shortness of breath (15.8% vs 5.4%) and headache (10.5% vs. 3.3%), by week 4. All analyses were also performed excluding cases that were reported as AI but were not confirmed by endocrinologists, with no significant differences in results.

To further analyze the co-occurrence of symptoms, heatmaps were generated with 32 symptoms that developed by week 6 to visualize symptom clusters (Fig. 3C). Diarrhea, vomiting, headache, fatigue and shortness of breath co-occurred in the majority of patients that later developed AI.

### Multiplicity of symptoms and irAE development

For each timepoint, a logistic regression was performed to determine if presence of a higher number of symptoms was associated with greater odds for later developing AI (Supplementary Table 5). At week 4, the odds of developing AI with each additional symptom increased by 1.46 (adjusted p-value = 0.008, mean number of symptoms in cases = 1.32 vs. controls = 0.75). At week 6, the odds of developing AI with each additional symptom decreased to 1.33 (adjusted p-value = 0.027, in cases = 1.65 vs. controls = 1.01). Number of symptoms was no longer a significant predictor by week 8 due to increasing number of symptoms in controls as well (adjusted p-value = 0.07, in cases = 1.83 vs. controls = 1.49).

### Early Symptoms Predictive of Hypothyroidism

Hypothyroidism was diagnosed at a median of 14 weeks (99 days) from treatment start (range = 20–208) which was close to the end of the immunotherapy phase of the treatment. Two patients developed hypothyroidism prior to week 4, and were censored from weeks 4,6 and 8 analyses, while an additional eight patients developed hypothyroidism between weeks 6 and 8 and were censored from the week 8 analyses.

For hypothyroidism, week 6 was also the most significant timepoint in the predictive model (adjusted p = 0.012) (Fig. 2B). Symptoms included in the OSB calculation (i.e. that were independently associated with hypothyroidism with p > 0.2) were painful urination, rash, shortness of breath and fatigue (Supplementary Table 3).

The Fig. 1B swimmers plot illustrates that higher-grade fatigue and rash were significantly enriched in the patients that later developed hypothyroidism. These symptoms presented earlier and lasted longer in those with immune related hypothyroidism. Multiple occurrences of rash, each relatively short duration, were more frequent, and fatigue was present for longer durations in patients with hypothyroidism. Shortness of breath was also more frequent and longer lasting in patients who subsequently developed hypothyroidism. These symptoms preceded the hypothyroidism diagnoses by an average of 57 days (approx. 8 weeks).

### Model Performance

A logistic regression was performed between the continuous OSB and outcome followed by.

The Youden’s best threshold OSB was 9.5 with a sensitivity of 0.43, specificity of 0.82 and accuracy of 0.77 (Supplementary Table 7 and Supplementary Figure S1B). As early as week 4, the mean OSB for patients that had hypothyroidism was more than two times higher than for patients that did not develop hypothyroidism (mean(SD) for case = 13.66(19.73) vs. control = 5.2(10.92)), and this gap widened greatly through week 8 (case = 14.6(22.66) and 20.64(37.21) vs control = 6.7(16.97) and 9.27(24.09) at weeks 6 and 8, respectively)(Fig. 2B, Supplementary Table 3).

### Model Validation

In the validation set of 217 patients receiving ICI with an oral paclitaxel backbone, 31 patients later developed hypothyroidism. Patients that later developed hypothyroidism had a higher mean OSB at week 6 than patients that did not develop hypothyroidism, though this difference was not statistically significant(13.5 vs 10). Model’s sensitivity was 16.1%, specificity was 84.4%, positive predictive value was 14.7%, negative predictive value was 85.8% and model accuracy was 74.7%.

### Longitudinal Symptom Enrichment

Binary analysis of symptoms showed that prior to week 4, fatigue was the only significantly enriched symptom among patients that later developed hypothyroidism(OR = 2.76, adjusted p = 0.047)(Table 2, Supplementary Table 4). At week 6, rash was the most significantly enriched symptom, followed by fatigue(OR = 3.09 and 2.87, p = 0.0037 and 0.011), and at week 8, only rash was significantly enriched in patients that later developed hypothyroidism(OR = 3.39, p = 0.0021). Fatigue was present in more than twice as many patients that developed hypothyroidism compared to those that did not at weeks 4(10.3% vs 4.0%) and 6(17.2% vs 6.8%)(Fig. 3B and Table 2); however, it was not significantly enriched in cases compared to controls at week 8, indicating that while early presence of fatigue can be an indicator of hypothyroidism, the growing incidence of fatigue among all patients undergoing treatment renders this an insignificant predictor as treatment progresses. Rash was present in twice as many patients at week 6 (20.7% vs 7.8%) and three times as many patients at week 8(24% vs 8.5%)(Fig. 3B and Table 2).

Further analysis and visualization of co-occurring symptoms revealed that diarrhea, rash, dizziness, and fatigue commonly co-occurred prior to week 6 among patients that later developed hypothyroidism(Fig. 3D). However, unlike for AI, the number of symptoms was not a significant predictor of later development of hypothyroidism(Supplementary Table 5).

## Discussion

ICIs are now a standard of care as neoadjuvant therapy combined with chemotherapy for stage II-III triple negative breast cancer, and for patients with PD-L1 positive metastatic TNBC. The current study utilized an analysis framework to identify unique symptom clusters that can be observed early on during ICI therapy and are predictive of developing the two most common irAEs, hypothyroidism and AI. Using prospectively collected clinical trial data from I-SPY2, we developed and validated a framework for early identification of patients at elevated risk for endocrine immune-related adverse events(irAEs). Our findings suggest that cumulative early symptom burden, especially by week 6 of treatment, provides meaningful predictive insight and could inform clinical strategies for early intervention. Identification of patients who are high risk for these irAEs could allow more frequent diagnostic testing (i.e. hormone level measurements) and proactive early treatment. While TSH is part of the standard of care monitoring, and cortisol level monitoring is now standard in I-SPY, by the time these tests are abnormal, neither AI nor hypothyroidism is likely reversible. Thus, the ability to find earlier indicators could potentially lead to proactive preventive interventions.

Overall, our results demonstrated that rash, shortness of breath, and fatigue commonly co-occurred among patients who later developed hypothyroidism (after a median of 57 days), while diarrhea, headache, vomiting, fatigue and shortness of breath were predictive of AI (after a median of 63 days). Dermatological symptoms, such as itching and rash, were also highly ranked symptoms in the first 4 to 8 weeks that were predictive for the development of hypothyroidism, while gastrointestinal symptoms, such as constipation and diarrhea, were predictive for the development of AI. Diarrhea often precedes constipation in both cases and controls, as symptom management for diarrhea can frequently lead to constipation in patients.

Fatigue remains one the most frequently reported side effects of cancer treatment and can be highly burdensome for patients. In the present study, fatigue that occurred early in treatment was a predictor of later development of hypothyroidism, however its significance dissipated as treatment continued and a high number of patients in both groups began to develop fatigue. This reinforces the need to consider symptoms longitudinally and in the context of other symptoms as markers of irAEs. Previous studies have found that early immune dysregulation, specifically with respect to cytokines associated with T-cell activation and autoimmune disease eventually lead to a discrete case of severe irAE[21]. Many of these early symptoms included in the model, such as diarrhea and constipation, fatigue, rash, headache, joint pain, etc. are symptoms that are commonly associated with immune dysregulation, thus it is possible that early immune dysregulation that ultimately leads to irAE development manifests through a combination of these symptoms.

We found that an overall symptom burden(OSB) score—capturing both severity and duration of early symptoms—was strongly associated with the later development of irAE, with a significant signal emerging as early as week 6 of therapy. Specific symptoms contributing to this burden included gastrointestinal (diarrhea, constipation, vomiting), respiratory (shortness of breath), and symptoms commonly associated with immune dysregulation (headache, joint pain, rash), These symptoms preceded the clinical diagnosis of AI by an average of nine weeks and hypothyroidism by 8 weeks, indicating a potential window for proactive monitoring and early evaluation. The consistency of these findings in an external validation cohort, despite different treatment backbones (oral paclitaxel rather than intravenous), reinforces the potential generalizability of this approach.

The results of this work can directly inform clinical care in several ways: 1) help clinicians risk-stratify follow-up and monitoring of patients at high-risk for toxicity; and 2) help develop and evaluate strategies to mitigate toxicities. Ultimately, this aids the overall goal of helping with clinical decision weighing patient toxicities against the benefit from immunotherapy. Through identifying those patients who are at highest risk, one can test de-escalation or supportive care strategies to decrease toxicity risk. Our models provide a strategy for early guidance and permit more personalized care by balancing increased efficacy with reduced risks of chronic and debilitating toxicities, as well as promoting early supportive care interventions that could mitigate the long -term damage that could result from these toxicities.

Previous studies examining ways by which to predict immunotherapy-related irAEs have focused on pre-treatment gene markers, host genetics, and circulating protein biomarkers including serum autoantibodies[22–25]. The current study utilizes a novel framework, leveraging early treatment-emergent symptoms in patients across 6 different immunotherapies, to determine risk of later development of serious irAEs. Early symptoms that we found to be enriched in patients with these irAE, provide clinicians a straightforward primary assessment tool in clinic for identifying patients that may have higher risk of these irAEs. The OSB, a sum of AUCs of selected symptoms, provides an assessment timepoint at week 6, where cumulative symptom burden in the early treatment phase provides a personalized risk estimates of the likelihood of later developing these key irAEs. Such a framework could pro-actively identify at-risk patients before the development of a serious irAE, with relatively minimal clinic and provider burden. The high specificity of our model would enable effective screening of patients that are at low risk of later irAEs and distinguishing them from those who might require additional monitoring or diagnostic interventions. Genetic markers predictive of predisposition to autoimmunity and patient reported symptoms have the potential to improve the predictive accuracy of our OSB score, which we are exploring. Patient reported outcomes might be particularly suited to strengthen this model as they can capture even earlier symptoms than the current assessments timed around clinic visits. Since June 2021, routine electronic patient reported outcomes data collection was launched at all I-SPY2 sites[26], and we are working on methodologies for incorporating real-time patient reported AE data with our CTCAE-symptom based irAE prediction model to further improve predictive power.

The clinical implications of our study, along with a growing evidence base regarding the adverse effects of immunotherapy, could enable more proactive management of patients to prevent these irAEs. The identification of symptoms (both early and those that persist), in combination with the identification of timepoints at which their emergence becomes most predictive, can make an important contribution to comprehensive survivorship care for women with breast cancer. Going forward, we will prospectively validate our methods in the ongoing I-SPY2.2 platform trial, as well as in other cancer trials and work to develop strategies to redirect therapy or develop preventive interventions when there are early indicators of treatment-induced long-term toxicities.

### Limitations

We analyzed data prospectively collected during a clinical trial, and the overall health and co-morbidity status of trial participants may not be representative of all patients with stage II/III breast cancer. All our patients received weekly paclitaxel, but the type of chemotherapy partner administered along with ICIs can influence the type and frequency of early symptoms. However, three of the six treatment arms used in this analysis included additional experimental therapies along with ICI and paclitaxel which suggest generalizability. Since many irAEs are rare, we could only analyze associations between early symptoms and subsequent irAE for the two most common AEs, hypothyroidism and AI. If early symptoms predict development of other irAEs remains unknown. Although our models performed well in the validation cohort, the positive predictive value remained modest, reflecting the low absolute incidence of these irAEs, and underscoring that symptom burden is likely only one piece of the risk puzzle.

## Conclusions

We show that a constellation of non-specific treatment emergent symptoms that develop as early as four to six weeks of ICI therapy could predict higher risk of future immune-related hypothyroidism and AI in patients with breast cancer. Since routine monitoring for AI with serial hormone level measurements is not currently recommended and the frequency of thyroid hormone assessments during therapy also vary, early onset fatigue, diarrhea, rash, shortness of breath could alert physicians to be more diligent in on-treatment and post-treatment monitoring for these endocrinopathies. Work is ongoing to develop strategies for monitoring and potential treatment mitigation to reduce the risk of permanent endocrinopathies.

## Supplementary Material

Supplementary Files

This is a list of supplementary files associated with this preprint. Click to download.

- SupplementaryTable1Armdetailsofpatients.xlsx
- SupplementaryTable2CTCAEitemsirae.xlsx
- SupplementaryTable3SymptomfeaturesforOSB.xlsx
- SupplementaryTable4enrichment.xlsx
- SupplementaryTable5LogisticRegressionofNumberofSymptomswithirAE.xlsx
- upSupplementaryTable6AdrenalThresholds.xlsx
- upSupplementaryTable7HypoThresholds.xlsx
- SupplementaryTable8Demographiccharacteristicsofvalidationset.xlsx
- SUPPLEMENTARYFIGURES.docx

## Figures and Tables

**Table 1:** Demographic characteristics of patient population by irAE type.

	Overall	Hypothyroidism (N = 61)	No Hypothyroidism (N = 421)	Adrenal insufficiency (N = 38)	No Adrenal insufficiency (N = 444)
**Age**					
Mean (SD)	47.6 (11.6)	46.4 (10.3)	47.8 (11.8)	48.5 (11.7)	47.6 (11.6)
Median [Min, Max]	47.3 [20.0, 79.0]	45.5 [28.8, 71.0]	47.9 [20.0, 79.0]	49.5 [31.0, 79.0]	47.0 [20.0, 76.0]
**Race**					
American Indian/ Alaskan Native	3 (0.6%)	1 (1.6%)	2 (0.5%)	0 (0%)	3 (0.7%)
Asian	32 (6.6%)	2 (3.3%)	30 (7.1%)	1 (2.6%)	31 (7.0%)
Black	60 (12.4%)	2 (3.3%)	58 (13.8%)	5 (13.2%)	55 (12.4%)
White	382 (79.3%)	53 (86.9%)	329 (78.1%)	32 (84.2%)	350 (78.8%)
**Ethnicity**					
Hispanic	78 (16.2%)	7 (11.5%)	71 (16.9%)	8 (21.1%)	70 (15.8%)
Not Hispanic	402 (83.4%)	54 (88.5%)	348 (82.7%)	30 (78.9%)	372 (83.8%)

**Table 2:** Binary (presence absence) enrichment analysis for symptoms. Symptoms with p-value < 0.2 at at least one timepoint are shown, ordered by p-value at week 4, with timepoints with adjusted p-value < 0.05 highlighted in green.

	WEEK 4				WEEK 6				WEEK 8			
ADRENAL INSUFFICIENCY												
Symptom	In Cases (N = 38)	In Control (N = 423)	Odds Ratio	Adjusted p-value	In Cases (N = 37)	In Control (N = 423)	Odds Ratio	Adjusted p-value	In Cases (N = 36)	In Control (N = 423)	Odds Ratio	Adjusted p-value
Diarrhea	18 (47.4%)	112 (26.5%)	2.49	0.0069	17 (45.9%)	131 (31%)	1.89	0.048	17 (47.2%)	143 (33.8%)	1.75	0.077
Constipation	2 (5.3%)	1 (0.2%)	23.03	0.019	2 (5.4%)	1 (0.2%)	23.68	0.018	3 (8.3%)	3 (0.7%)	12.56	0.0075
Shortness of breath	6 (15.8%)	23 (5.4%)	3.25	0.024	7 (18.9%)	37 (8.7%)	2.43	0.051	6 (16.7%)	41 (9.7%)	1.86	0.15
Headache	4 (10.5%)	14 (3.3%)	3.42	0.052	4 (10.8%)	17 (4%)	2.88	0.079	4 (11.1%)	20 (4.7%)	2.51	0.11
Palpitations	2 (5.3%)	4 (0.9%)	5.78	0.08	2 (5.4%)	7 (1.7%)	3.38	0.16	3 (8.3%)	8 (1.9%)	4.69	0.047
Fatigue	4 (10.5%)	18 (4.3%)	2.64	0.097	6 (16.2%)	31 (7.3%)	2.44	0.065	5 (13.9%)	49 (11.6%)	1.23	0.42
Taste changes	1 (2.6%)	2 (0.5%)	5.65	0.23	2 (5.4%)	2 (0.5%)	11.88	0.034	2 (5.6%)	2 (0.5%)	12.22	0.032
Dizziness	3 (7.9%)	29 (6.9%)	1.16	0.5	6 (16.2%)	39 (9.2%)	1.9	0.14	6 (16.7%)	46 (10.9%)	1.64	0.21
HYPOTHYROIDISM												
Symptom	In Cases (N = 58)	In Control (N = 400)	Odds Ratio	Adjusted p-value	In Cases (N = 58)	In Control (N = 400)	Odds Ratio	Adjusted p-value	In Cases (N = 50)	In Control (N = 400)	Odds Ratio	Adjusted p-value
Fatigue	6 (10.3%)	16 (4%)	2.76	0.047	10 (17.2%)	27 (6.8%)	2.87	0.011	8 (16%)	44 (11%)	1.54	0.2
Rash	7 (12.1%)	22 (5.5%)	2.35	0.059	12 (20.7%)	31 (7.8%)	3.09	0.0037	12 (24%)	34 (8.5%)	3.39	0.0021
Itching	2 (3.4%)	4 (1%)	3.52	0.17	3 (5.2%)	6 (1.5%)	3.57	0.093	3 (6%)	8 (2%)	3.12	0.11
Shortness of breath	5 (8.6%)	24 (6%)	1.48	0.3	9 (15.5%)	36 (9%)	1.85	0.097	6 (12%)	39 (9.8%)	1.26	0.38
A)												

## Data Availability

Subject-level data for this study is available to approved investigators completing a request form available at: https://www.quantumleaphealth.org/for-investigators/clinicians-proposal-submissions/.
